# Potassium and methionine mitigate the alternate bearing of Balady mandarin via reducing gibberellins and increasing salicylic acid and auxins

**DOI:** 10.1038/s41598-025-04943-z

**Published:** 2025-06-20

**Authors:** Ayman EA Shaban, Mohammed IM El-Banna, Ahmed AbdelHady Rashedy

**Affiliations:** Pomology department, Faculty of Agriculture, Cairo University, Giza Governorate, Egypt

**Keywords:** *Citrus reticulate*, Mandarin, Yield, Carotenoids, Fruit quality, Sugars, Biochemistry, Ecology, Physiology, Plant sciences

## Abstract

Alternate bearing (AB) is a major challenge for citrus orchards. Increasing yield in one season (On year) leads to more seeds (fruits) as a supplier of gibberellins, which delay harvesting with low fruit quality and also decreases flowering and fruiting in the next season (Off year). So, the aim of this study is improving Balady mandarin fruit quality as well as accelerating fruit harvesting during the On year via foliar nourishments with potassium citrate (KC) or nitrate (KN) at 0.5% incorporated with either methionine (M) at 0.2% or sulphur (S) at 0.3%, twice two months before harvest (at flower bud induction time). The results indicated that, during On year, all treatments accelerate harvesting date, improve fruit weight, yield, fruit quality with a highly significant effect for KC + M treatment compared to the control. Moreover, this treatment increased fruit yield by 20.72% and 33.74%, for the first and the second On years, respectively. The most promising effect for KC + M is decreasing gibberellins levels during December (flower bud induction) in On year by 7& 19.4% and during January (before flowering) in Off year by 19.4% and 17.44%. Moreover, it increased both salicylic acid and auxin in the following Off year (before flowering) by 17.44%, 42.9 and 40%, respectively. This findings led to increase fruit number by 272.64% and 267.94%, and fruit yield by 251.3% and 289.65% for the following two off-year as well as decreasing AB by 61.7% and 61.67%. This study highlights the efficiency role of KC as a key for improving fruit quality during On year where heavy fruit load, as well as M application for overcoming AB as anti-gibberellins agent via accelerating harvesting in On year and enhances flowering in the following Off year via hormonal control.

## Introduction

Citrus fruits, including mandarins are widely cultivated in Egypt, accounting for 25% of the country’s citrus output^1,2^. Balady mandarin (*Citrus reticulata*, Blanco) cultivation covers 24.6% of Egypt’s total citrus area, with production reaching 1,174,895 tons in 2022^3^ Mandarins are popular in Egypt for their high yield, easy peeling, and good juice quality. They are rich in essential vitamins, antioxidants, and minerals^4^.

Alternate bearing (AB) is a pattern in tree species, involving heavy fruit production in one year referred to as On year and reduced yield or no fruit in the next known as Off year^5^. It affects agricultural practices and ecological dynamics. Factors influencing AB include high seed counts, late harvest timing, high carbohydrate consumption, and gibberellin acid production which is known as inhibitor of flowering in citrus^6^.

AB in citrus production is a significant challenge for producers. Balancing nutrition is crucial for reducing this phenomena^7^. Potassium (K) is essential nutrient for citrus growth and production, increasing fruit size, vigor, and taste^8,9^. K deficiency can lead to poor yield and quality, affecting the industry’s sustainable development. A balanced K dose is vital for maximum fruit crop output, as growth, yield, and fruit quality vary depending on K sources, levels, and application frequency^10,11^. Farmers widely use K fertilizers such as potassium sulfate, potassium nitrate, potassium chloride, and potassium carbonate for crops, especially in Egypt where clay soils trap K^12^. Rising prices of K fertilizers are a challenge for small farmers^13^. Thus, finding better ways to use K is an important practice. One effective method is applying foliar K fertilizers directly on leaves, which is cost-effective, more efficient, and can lead to better yields^14^. There is an urgent need to find alternative K sources and optimal doses to enhance citrus quality and yield^15^.

Sulphur (S) is crucial for the stability and production of photosynthetic pigments and plant growth under varying environmental conditions^16,17^. Application of S improves physiological processes in plants by augmenting photosynthetic pigment biosynthesis as carotenoids, enzymatic activities and photosynthetic attributes^18,19^.

Methionine (M) is an essential amino acid that is safe, inexpensive, and simple to use^20^. In addition, studies have reported that M also has a wide range of functions in plants, such as participating in plant systemic defences and reducing the incidence of plant-infectious diseases^21,22^. M is considered the main precursor of ethylene biosynthesis in higher plants through a pathway involving S-Adenosyl-Methionine and l-Aminocyclopropane-l-Carboxylic Acid^23^. Studies have also reported that M treatment is beneficial in maintaining the biochemical and sensory quality of different fruits and vegetables^24,25^.

In Egypt, like subtropical regions, flower bud induction occurs in winter typically January^26–28^. Plant hormones regulate citrus flowering; gibberellins promote vegetative growth^29,30^ but inhibit flowering^31,32^. On the other hand, the recent studies proved that, salicylic acid is essential for flower formation and development^33–36^, while fruit load can inhibit inflorescence growth by selectively dampening auxin transport in the apical stem region^37^.

The objective of this study was to determine the impact of foliar application of potassium citrate (KC) or potassium nitrate KN combined with sulphur (S) or methionine (M) on AB patterns, yield and fruit quality of Balady mandarin trees during On year and the yield component in the following Off year.

## Materials and methods

### Experimental site

The authors identify that the institutional and/or licensing committee that approved the experiments, including any relevant details, confirm that all experiments were performed in accordance with relevant named guidelines and regulations.

The present study was carried out during 2021, 2022 and 2023 seasons (two On year & two Off year seasons) at a private orchard at El-Nagah district (30°41’42"N and 30°23’16"E, elevation 9 m), El-Beheira Governorate, Egypt.

### Plant material and treatments

Twelve years-old Balady mandarin (*Citrus reticulate* Blanco) trees budded on sour orange (*Citrus aurantium* L.) rootstock and spaced 5 × 5 m apart. Trees were grown in sandy soil under drip irrigation system. Potassium citrate (KC) or potassium nitrate (KN) was incorporated with methionine (M) and sulphur (S) as the following. Control (untreated trees), KC + S, KC + M, KN + S and KN + M. All materials were applied as foliar spraying twice at 15 September and 15 October early in the morning. KC and KN used at 0.5% while M at 0.2% and S at 0.3%. All sprayed combination materials added together immediately before spraying. All trees received the horticultural practices (irrigation, fertilizers, pruning, weed control, pest control …etc) according to recommendation of Agriculture Ministry.

The plant sources Balady mandarin (*Citrus reticulate* Blanco) and sour orange (*Citrus aurantium* L.) were produced from the Agricultural Research Station, Faculty of Agriculture, Cairo University, Egypt (Latitude: 30° 01’ 39.36” N; Longitude: 31° 12’ 36.50” E). Also, The necessary permits and licenses have been obtained to grow oranges in the orchard in the Al-Najah area.

## Measurements

### Harvest date

Harvest date was recorded for each treatment separately to determine effect of treatments on early harvesting. Samples was taken one month before harvest to determine the suitable ripening stage according to TSS/acid ratio, fruit weight and color.

### Yield component

At harvest time, yield of each tree was determined as number and weight (kg) of fruits/tree during On and the following Off year. Fruit weight was recorded through digital sensitive balance.

### Alternate bearing (AB) index

It is determined according to Jangid et al.^38^ as the following equation:

$$((On \; year\;yield - the following\ Off\;year\;yield)\div(On\;year\;yield + the following\ Off\;year\;yield))$$

## Fruit quality

### Total soluble solids, titratable acidity

Fruits were cut in half and carefully hand-squeezed in a commercial juicer. The content of total soluble solids (TSS) was determined through a handheld refractometer (model MASTER-53 S; Atago Co. Ltd., Tokyo, Japan) and expressed as °Brix. Titratable acidity (TA) was determined by titrating 10 mL juice to the end point of pH 8.1 with 0.1 N NaOH and expressed as a percentage of citric acid. Once the TSS and TA contents had been assessed, the ripening index was calculated as the TSS/TA ratio^39^.

### Total sugars content

Total sugar content was determined using the phenol–sulfuric acid method^40^. Fruit pulp samples (0.25 g—collected from three fruits) were homogenized in 20 mL of 70% ethanol (v/v) and filtered. The filtrates (1 mL) were treated with 1 mL of 5% phenol (v/v) and 5 mL of 98% sulfuric acid (v/v). After 1 h, the absorbance of the cold and colored solutions was read at 490 nm using UV/Vis spectrophotometer (UNICO S2100, Cole Parmer Instruments, Chicago, IL, USA). A standard curve was generated using a standard glucose solution, and total sugar content was expressed as mg glucose equivalents per g fresh weight.

### Total carotenoid content

Fruit peel samples (0.25 g—collected from three fruits) were homogenized in 20 mL of 80% acetone (*v*/*v*) for carotenoid extraction. Extracts were filtered and absorbance was measured using a UV/Vis spectrophotometer (UNICO S2100, Cole Parmer Instruments, Chicago, IL, USA) at 480 and 510 nm. Total carotenoid content was calculated according to Jensen^41^ as µg·g^−1^ of fresh weight using the following formula:

$$Total\;Carotenoids\;(\mu g \cdot g^{-1}) = (7.6\times OD\;480) - ((1.49 \times OD\; 510) \times (V/1000) \times W)$$

where OD = optical density, V = final volume of 80% acetone, and W = sample weight.

### Ascorbic acid content (Asc)

The content of ascorbic acid Asc in the juice was determined following the method described earlier by AOAC^42^ (1990). Briefly, 10 mL of each sample juice and 90 mL of oxalic acid solution (0.4%) were homogenized and filtered with Whatman No. 2 filter paper. A 5 mL aliquot was titrated to the newly prepared 2,6-dichlorophenolindophenol dye to a light pink endpoint. By using the given below formula, Asc contents were calculated and expressed as mg 100 mL^−1^ juice.

$$Asc\;content = ((1 \times R1 \times V \times 100)\div(R \times W \times V1))$$

where R1 = mL of dye used to titrate against aliquot V1 (sample reading); V = volume of the aliquot made by 0.4% oxalic acid; R = mL of dye used to titrate against 1.0 mL of reference solution (standard reading); W = mL of juice used; and V1 = mL of aliquot used for titration.

### Plant hormone

For both seasons, Gibberellins samples were taken from treated trees(leaves) at the mid of November (flower bud induction) after one month after treatments (On year) and also at January (two months before flowering) in Off year trees of the same tree. while, in the second season, salicylic acid and indole butyric acid samples were taken from leaves of treated trees at January (two months before flowering) in Off year trees of the same tree. All samples were analysed using HPLC.

The collected samples were analysed using Agilent1260 infinity HPLC Series (Agilent^®^, USA), equipped with Quaternary pump, a Zorbex Eclipse Plus C18 column 100 mm x 4.6 mm (Agilent^®^, USA), operated at 35^o^C. The separation is achieved using gradient elution with (A) 5 mM Ammonium acetate/0.05% formic acid in water (B) Acetonitrile. The injected volume was 20 µL. Detection: Variable Wavelength Detector set at 254 nm.

### Statistical analysis

The experiment was arranged in a complete randomized design with three replicates, each consisted of four trees. Results were subjected to an analysis of variance (ANOVA) using the general linear model (GLM) procedure—SAS software Version 9.0 (SAS Institute Inc., Cary, NC, USA). Mean comparisons between treatments were performed using Duncan’s multiple range test^43^ at a significant level of *p* < 0.05.

## Results and discussion

### Harvest date

**Fig. 1:**
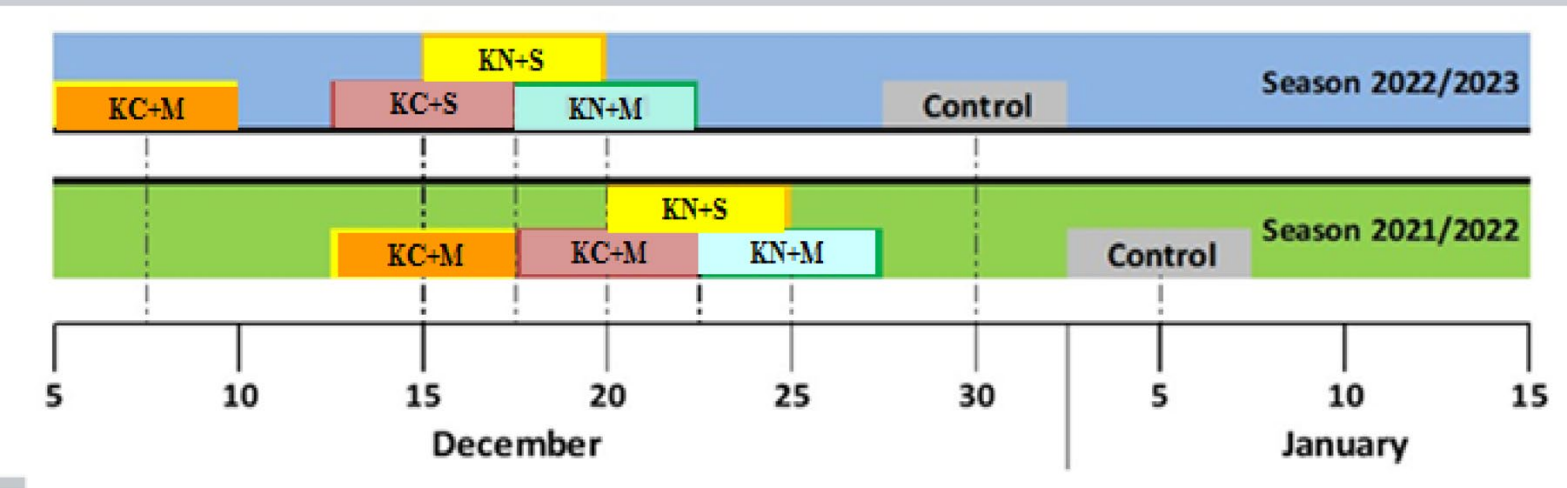
Effect of potassium form (0.5%), methionine (0.2%) and sulphur (0.3%) on harvesting date of Balady mandarin fruits. PC = potassium citrate, PN = Potassium Nitrate, S = Sulphur, and M = Methionine.

The positive effect of foliar spraying of K fertilizer combined with S or M on harvesting date was observed (Fig. 1). All treatments led to early harvesting of Balday mandarin fruits compared to untreated fruits by 10–20 days in 2021 season and 10–23 days in season 2022. The early harvested fruits were that sprayed with KC + M with 20–23 days depend on season earlier than untreated fruits, while control fruits were the last to harvest among different treatments. Earliness in fruit harvesting may be due to reach maturity and ripening and become ready for consumption through spraying with different K fertilizers and M or S compared to untreated trees.

### Yield components

#### Fruit number per tree

**Fig. 2:**
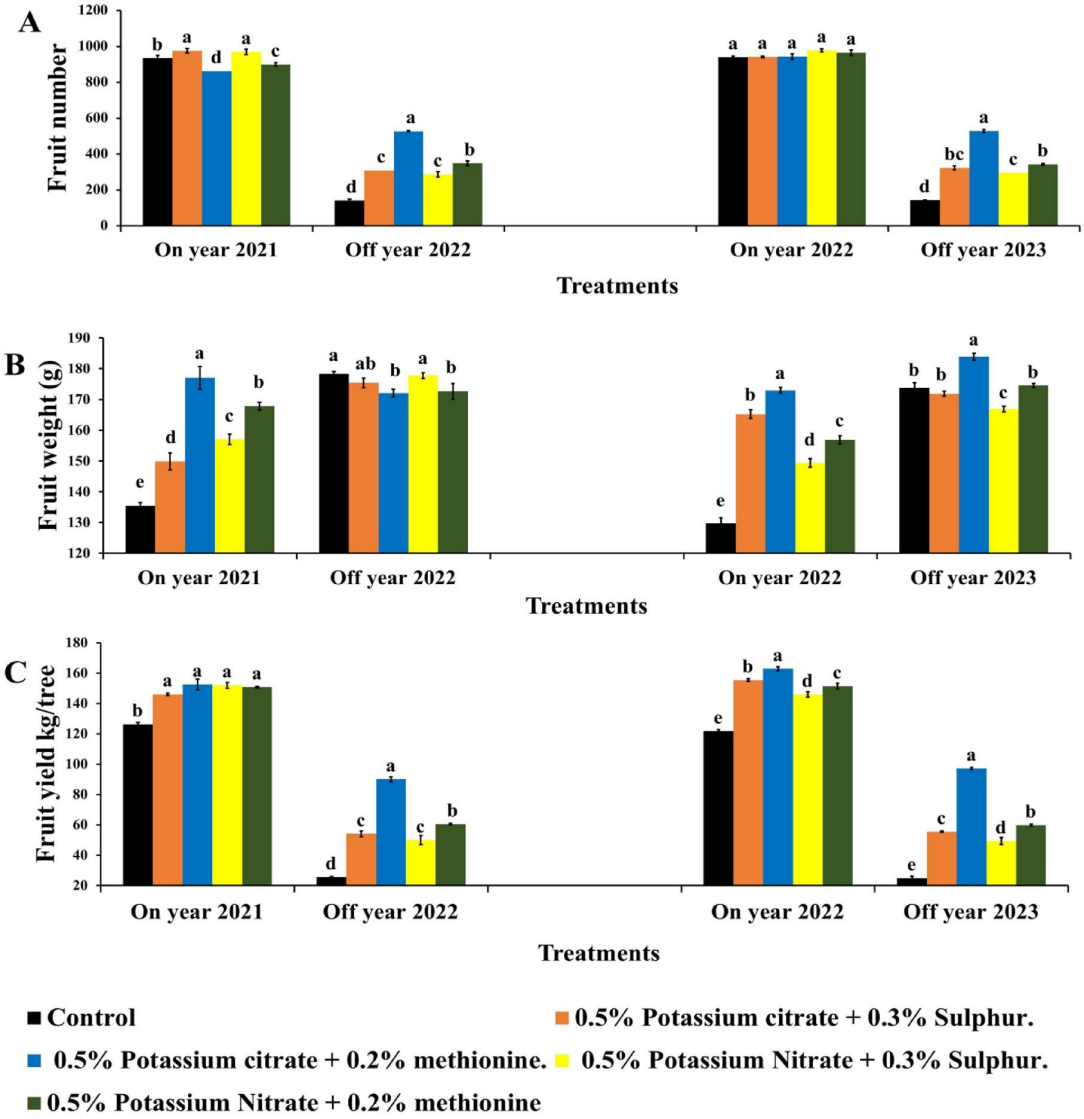
Effect of foliar application of potassium citrate or nitrate at 0.5% combined with either methionine (0.2%) or sulphur (0.3%) on fruit number (**A**), fruit weight (**B**) and fruit yield (**C**) of Balady mandarin fruits during On and the following Off year through three seasons. Data were presented as means (*n* = 3 ± SE).

Spraying K fertilizers combined with S or M had no effect on fruit number of Balady mandarin trees during On year in the second season (Fig. 2A). This may be due to the fruit number was determined early during fruit set and there is no effect of the used substances on increasing fruit drop during the late stage. However, there were significant differences between all treatments and control during Off year in both seasons (Fig. 2A). KC + M as foliar application on Balady mandarin trees in On year resulted in highest fruit load of trees (525 fruits/tree in average) followed by KN + M (345 fruits/tree in average) during Off year, which increased by 272.6% and 267.9% than the control in both seasons, while the increase in On year was − 7.67& 0.43%. Untreated trees showed lowest fruit number per tree in both Off year seasons (142&144 fruits/tree in average). Increasing fruit number was higher in off year in treated trees compared to the control may be due to decreasing GA levels and early harvesting which decrease the consumption period of carbohydrates and nutrients.

The seeds of fruit have an inhibit effect on the following flower bud induction and flowering^44–46^. Since seeds produce more GA during flower bud induction period which decreased it^6^. GA levels become high then low during the On and the following Off year, respectively which subsequently had negative or positive effects on the flowering process^47^. Exogenous GA application during flower bud induction reduced flowering intensity^48,49^. This is similar to high fruit load during On year season^45^.

It seems that early harvesting of fruits during On year due to spraying K fertilizers combined with S or M had a great effect on AB. Advancing harvest date of fruits (source of GA_3_) decrease inhibitory effect of GA_3_ during flower bud induction period and resulted in decreasing AB of Balady mandarin trees during Off year.

### Fruit weight (g)

K treatments increased the fruit weight over control (Fig. 2B) and it was also observed that there was an increase in fruit weight depend on combined S or M. KC + M significantly increased fruit weight during On year by weight by 30.71((172.95 g) and 33.24% (177 g) in season 2021 and in season 2022 compared to the control (135.42&129.81 g). During Off year KC + M increased fruit weight by −3.5% and 5.86% than the control. The rate of increasing fruit weight was higher in On year than Off year may be due to higher yield (fruit number) in treated trees compared to the control which provide to distribute growth substances, water, carbohydrates to more number of fruit. While in the same (On year) K and M or S consumed by the same amount of fruits.

Spraying Balady mandarin trees with K, which consider a fruit quality key, had a positive effects on fruit quality (fruit weight, number of fruits) and finally fruit yield^11^. Spraying K increased fruit size of Kinnow fruit^50,51^. Also, combining KN, phosphate and humate increased fruit weight of sweet lime^52^. And increased Clementine fruit firmness and rind thichness^53^.

Soil application of K fertilizers had lower effect than foliar application in different soil types^54,55^. This effect may be due to high mobility of K for the growing fruits which increase water storage and regulate osmotic process^56,57^.

### Yield (Kg/tree)

Foliar application of K fertilizers increased the yield of Balady mandarin trees in both seasons even in On or Off year (Fig. 2C). It is resulted from improving fruit weight due to spraying K fertilizers reflected on fruit weight then total yield during On year. Moreover, increasing yield of Off trees was correlated with the main increase in fruit number. KC combined with methionine M recorded the highest yield in On (about 152–163 Kg/tree) and Off (90–97 kg/tree) trees in both seasons this increase recorded by 20.72% & 33.74% during the On year and 251.30% & 289.65% during Off year compared to the control. Increase yield in Off trees as indirect result of spraying K fertilizers compared to control reflect its role in management of (AB) alternate bearing besides decreasing GA levels due to early harvesting and M methionine as anti-gibberellins.

Generally, the next Off year showed the most promising effect for KC + M increased fruit number by 272.64% & 267.94% and fruit yield by 251.3% & 289.65%. By contrast, the increases during On year recorded of −7.67% & 0.43% and 20.72% & 33.74%, respectively. In this respect, Vijay et al.^56^ indicated that spraying Jaffa sweet orange with KN and sulphate improved fruit weight and increasing yield. This increasing in fruit yield due to KN treatment was correlated with the increases in fruit weight^58^. These results were in agreement with Shen et al.^51^ who reported that trees treated Kousui Japanese pear with KNO_3_ significantly increase yield. K is the most crucial macro-nutrient for citrus fruits, depending on its important role in improving yield and fruit quality^59–61^.

### Total following on + Off year yield

**Fig. 3:**
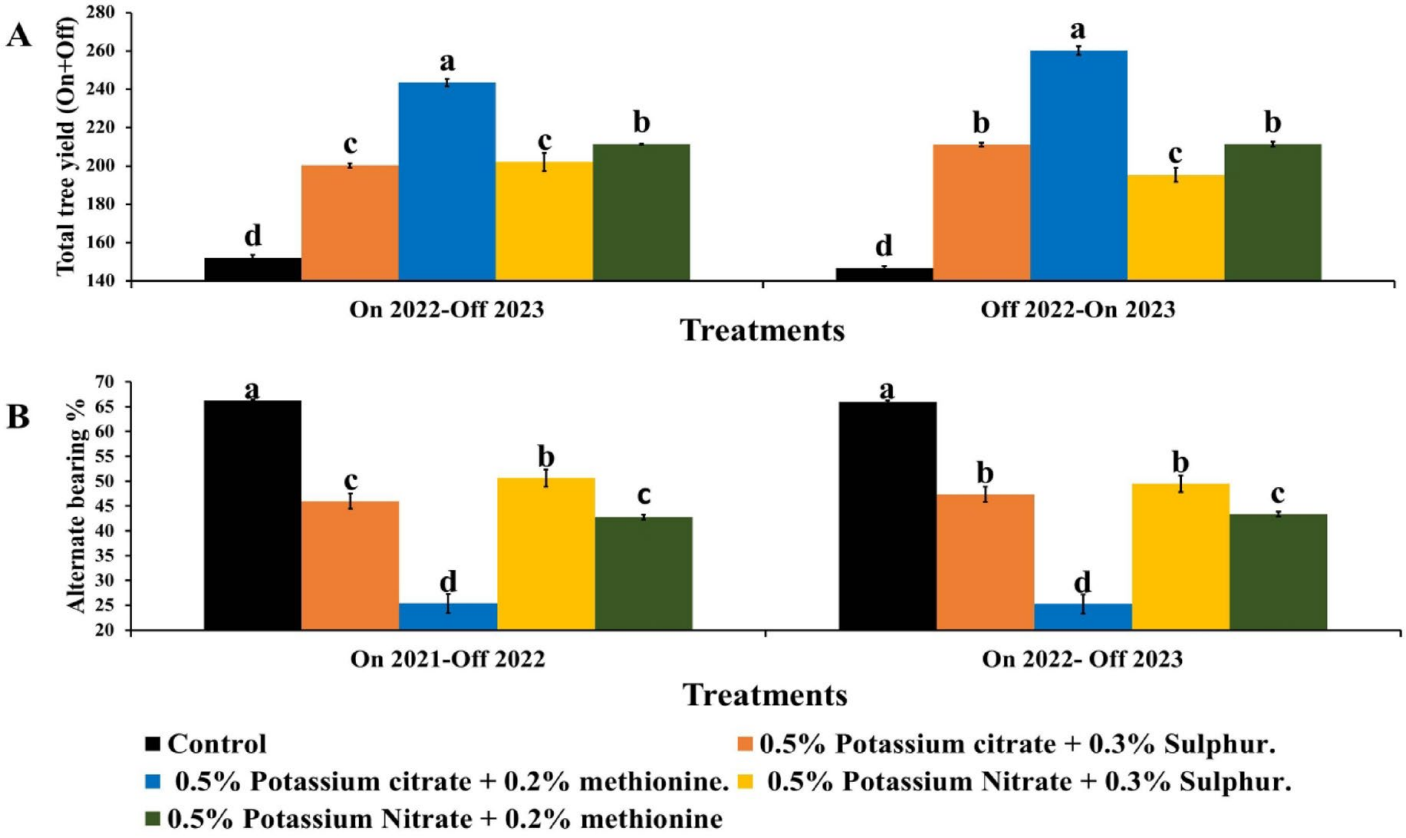
Effect of foliar application of potassium citrate or nitrate at 0.5% combined with either methionine (0.2%) or sulphur (0.3%) on total following On + Off year (**A**) and alternate bearing (**B**) of Balady mandarin fruits. Data were presented as means (*n* = 3 ± SE).

All treatments increased total On + Off yield significantly compared to the control (Fig. 3A). Also, KC + M recorded the highest total yield (On + Off) by 60&77.2% followed by KN + M 39&44% more yield than the control. This increase was due to improve fruit weight in On year as well as improve fruit set, fruit number and weight in the following Off year.

#### Alternate bearing (AB) index

Figure 3B show the effect of two forms of KC or KN incorporated with M & S on AB of Balady mandarin trees. It can be noticed that, both KC or KN succeeded in decreasing AB either combined with M or S. Moreover, KC + M recorded the lowest significant AB by 61.70 and 61.74%, followed by KN which decreased AB by 35.49 and 34.37% than control which recorded the highest significant AB (66.27&66%). The reduction in AB due to K and M treatments resulted from the fact that, first K improve fruit weight during On year. Second, M accelerate fruit harvest during On year which consider the higher supplier with gibberellins the anti-flowering hormone. Third, M consider biosynthesis ethylene which stimulate flowering in the following year (Off year). These results were in line with Monselise and Goldschmidt^6^ who found high seeds number, carbohydrates consumption, and gibberellins production the most important reasons for AB bearing. These results were in line with Stander et al.^62^ as they reported that during On year ‘Nadorcott’ Mandarin fruit load restrict summer vegetative growth and restrict roots growth which decrease flowering in the following Off year by disturb the balance between root growth and vegetative shoot development by limiting carbohydrate allocation to roots. Also, Abobatta^5^ suggested spraying with GA to management aalternative bearing in citrus varieties before On year.

### Fruit quality

#### TSS, acidity and tss/acidity ratio

**Fig. 4:**
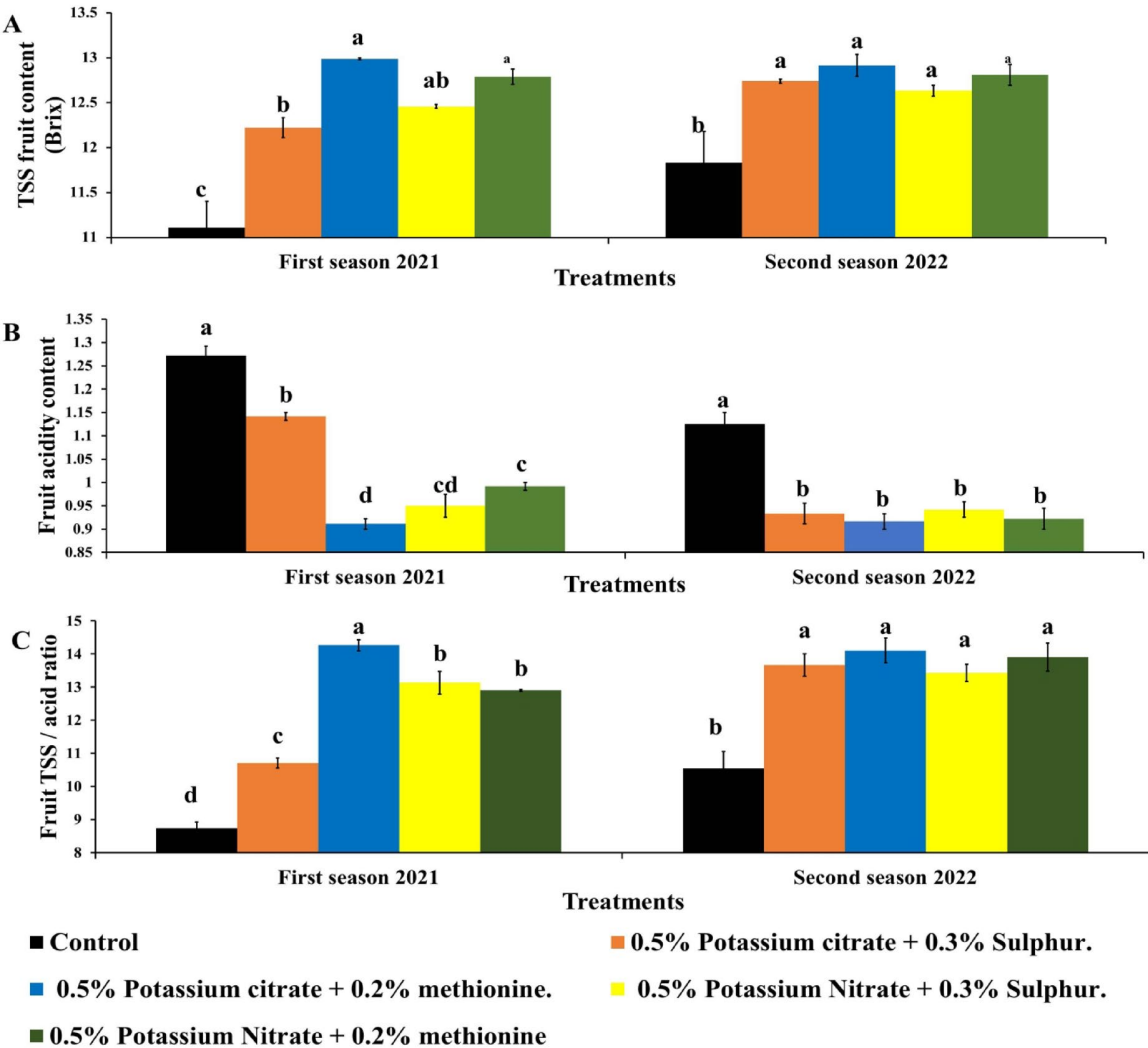
Effect of foliar application of potassium citrate or nitrate at 0.5% combined with either methionine (0.2%) or sulphur (0.3%) on fruit TSS (**A**), fruit acidity content (**B**) and fruit TSS/acid ratio (**C**) of Balady mandarin fruits during two seasons. Data were presented as means (*n* = 3 ± SE).

The TSS content of Balady mandarin fruits varied noticeably among treatments (Fig. 4A). The maximum TSS content in fruit was obtained in the trees sprayed with K in 2021 season. All treatments had same degree of significant increase compared to control in season 2022. Control (untreated) fruits showed minimum TSS content in both seasons (11.11–11.84 °Brix). Generally, KC + M increased TSS by 10% & 7.67% and TSS/Acid by 10%&7.68% in the first and second seasons, respectively. Fruit acidity was significantly lowered in all treatments compared to control (Fig. 4B). Fruits sprayed with KC + M recorded the lowest value of acidity (0.91%) in season 2021. In comparison, the highest TA value (1.13–1.27%) was observed in control fruits over the rest of the treatments depend on season. Regarding the effect of K foliar treatments combined with S or M on the TSS: TA ratio (Fig. 4C), the maximum ratio (14.26) resulted from spraying of KC + M in season 2021. All treatments increased TSS: TA ratio compared to untreated fruits in both seasons. These results indicated TSS: TA ratio could be enhanced by spraying different K fertilizers on Balady mandarin fruits.

Foliar application of K regardless the source used, increased fruit total soluble solids which associated to K application^53^. Many recent studies have shown that K is the main controlling element for improving citrus fruit qualities like fruit size, soluble solids and total fruit acidity^59,60^. The maximum TSS content in fruit was obtained in the trees sprayed with K_2_SO_4_ throughout the fruit development. Spraying K resulted in a significant reduction if fruit acidity. Also, it led to improving TSS: TA ratio of tangarin fruits^63^. K stimulates cell division, cell elongation and root growth which support plant growth and nutrients uptakes^60,64^. Moreover, K stimulated translocation of sugars from production organs to storage fruit which increased fruit TSS content^65^.

#### Total sugars

K fertilizer source combined with M or S had a slight effect on sugar content of mandarin fruit (Table 1). KC + M recorded the highest values in total sugar content with increase about 19% followed by KN + S in 2021 season compared to control. Total sugars two seasons by 19.43 and 5.1% in first and second seasons, respectively.

Gill et al.^66^ observed in Kinnow mandarin that various K formulations and spray schedule did not influence the total sugar content of juice. Similarly, Sangwan et al.^67^ found that sugar content of the juice did not vary significantly with various K treatments. The contents of different soluble sugars were enhanced in K_2_SO_4_-treated juice sacs of Nanfeng tangerine fruits^63^.

**Table 1:** Effect of potassium citrate (KC)and potassium nitrate (KN) combined with sulphur (S) or methionine (M) on total sugars, total carotenoids and ascorbic acid C content of Balady Mandarin fruits during two seasons.

Treatments	Total sugars content %		Total carotenoids (mg/100 g fresh weight)		Ascorbic acid C (mg/100 ml juice)	
	2021	2022	2021	2022	2021	2022
Control	7.72^d^	8.64^a^	7.07^c^	6.58^c^	38.78^b^	38.00^b^
0.5% KC + S	8.33^c^	8.05^b^	7.41^bc^	6.87^c^	39.39^b^	39.08^b^
0.5% KC + M	9.22^a^	9.08^a^	8.94^a^	8.67^a^	42.22^a^	41.41^a^
0.5% KN + S	8.66^b^	8.92^a^	7.92^b^	7.50^b^	39.33^b^	38.92^b^
0.5% KN + M	8.36^c^	8.83^a^	7.96^b^	8.44^a^	38.83^b^	39.11^b^

KC = potassium citrate, KN = potassium nitrate, M = methionine and S = sulphur.

#### Total carotenoids content

The results indicated that spraying K fertilizers with addition of S or M is an effective approach to improving the peel colouration (Table 1). Using KC + M as foliar application showed the highest content of carotenoids in peel with increase about 26 & 31% in the first and second season, respectively. Addition of M to K source was found better than S in enhancing Balady mandarin color. Poor coloration was observed in control fruits compared to other treatments due to significant differences in carotenoids content.

Degradation of chlorophyll contents and accumulation of carotenoids are important fruit maturity indices in citrus fruits^68^. Spraying K fertilizers, in particular K_2_SO_4_, is an effective approach to improving the peel pigmentation. The results showed that spraying K_2_SO_4_ across the whole fruit development can enhance peel coloration with a higher ratio of concentrations of carotenoids relative to chlorophyll^63^. This indicated that K_2_SO_4_ is essential for enhancing the synthesis of carotenoids and promoting fruit color preference^69^.

### Asc content

Asc content was not affected by different treatments (Table 1). There no significant differences were observed except for application of KC + M which recorded high Asc content by 8.8 and 9% in the first and second seasons, respectively.

Asc of fruits was enhanced by Various K formulations as compared to control. Among the various K sources, KCl proved better in improving the Asc content^66^. Sangwan et al.^67^ observed maximum Asc with KNO_3_ treatment. Application of K recorded maximum a Asc of juice^70^. Sarrwy et al.^71^ found that spraying KNO_3_ combined with 0.5% chelated Zn recorded the highest significant values of Asc percentages compared with other treatments in Balady mandarin. Recent reports indicated that K had important role in enhancing Asc^60^. However, Mostafa et al.^72^ and Mostafa and Saleh^11^ reported that spraying various citrus varieties with different K forms did not affect the Asc. Increased Asc with foliar application of K might be due to the role of K in activating the synthesis of Asc somewhere between D-Glucose to L-Ascorbate^73^.

### Plant hormone

**Fig. 5:**
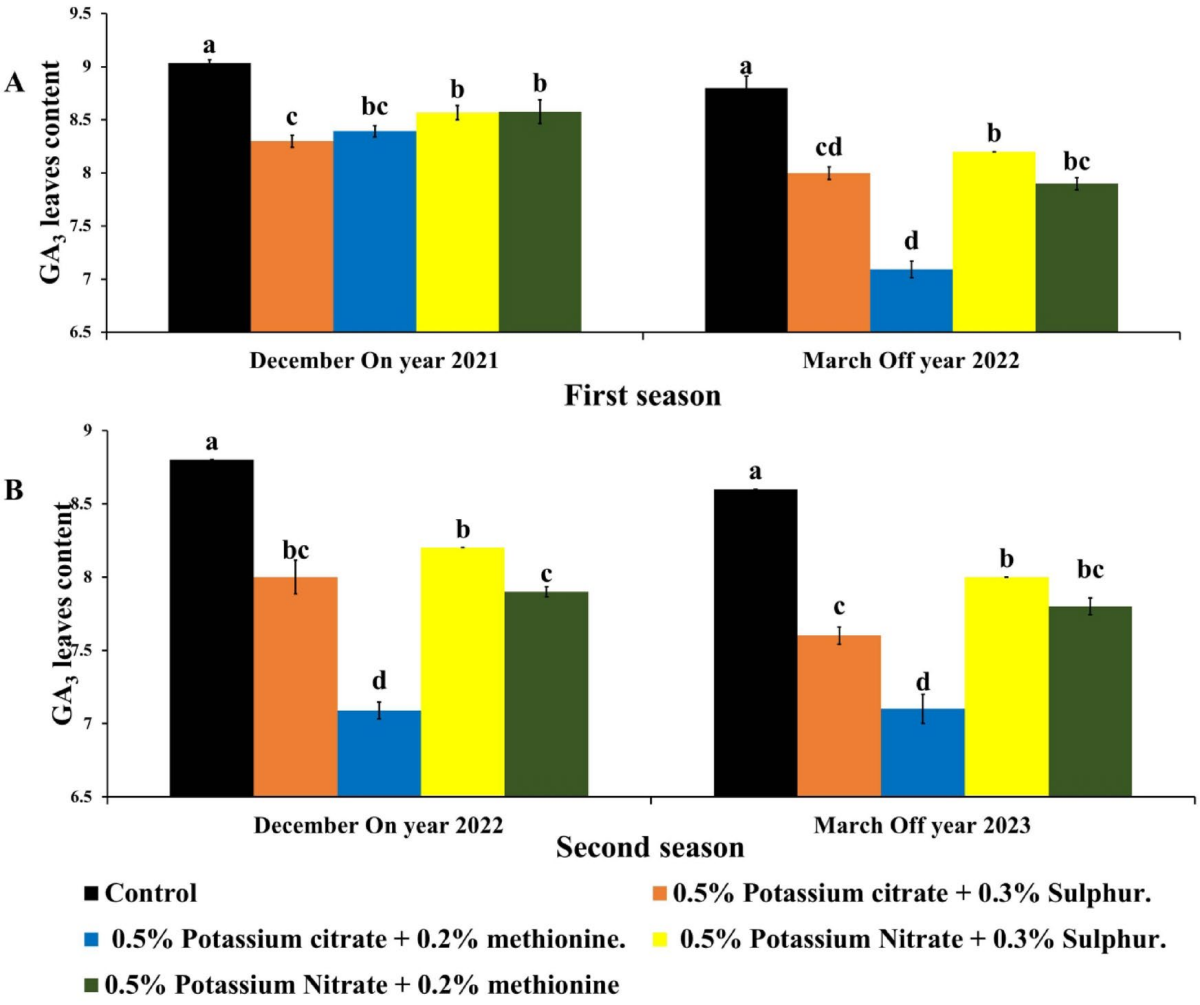
Effect of foliar potassium citrate or nitrate at 0.5% combined with either methionine (0.2%) or sulphur (0.3%) on gibberellins content of Balady mandarin fruits during On and the following Off year for two seasons. Data were presented as means (*n* = 3 ± SE).

Foliar application of K fertilizers with M methionine or (S)sulphur has a positive effect on inhibitory effect of GA_3_ on citrus flowering (Fig. 5). Early harvesting of Balady mandarin fruits during On year due to K treatments resulted in decrease GA_3_ two months before flowering. Highest GA_3_ levels were observed in untreated trees (about 8.6–8.8 µg/g fresh weight) because of delaying of harvest date which explain inhibitory effect on flowering and yield of trees during the following Off years. KC + M or KC + S resulted in early harvesting with 20–25 days earlier than untreated trees which reflected on GA_3_ content in leaves. Lowest GA_3_ content in leaves was found in trees sprayed with KC + M which recorded decreasing GA levels by 9.4%&17.44%.

The most promising effect for (KC) potassium citrate incorporated with methionine M is decreasing leaves gibberellins levels during December (flower bud induction) in On year by 7& 19.4% and during march (before Off year flowering in the following Off year by 19.4%&17.44% which led to increase fruit number by 272.64 & 267.94%, and fruit yield by 251.3% & 289.65% for the following two off-year.

The seeds of fruit have an inhibit effect on the following flower bud induction and flowering due to more GA production during flower bud induction reduced flower intensity^44–46^.

**Fig. 6:**
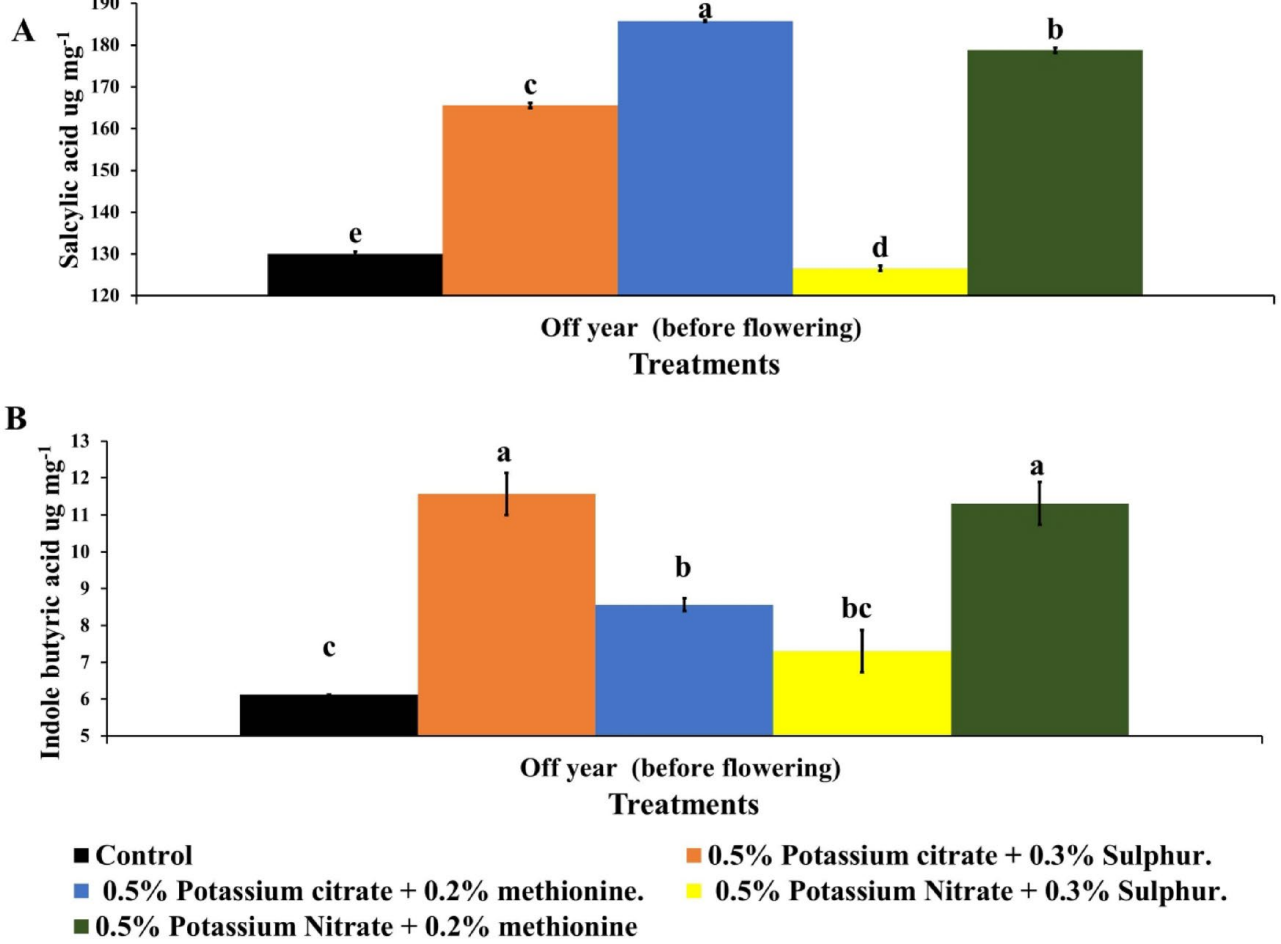
Effect of foliar potassium citrate or nitrate at 0.5% combined with either methionine (0.2%) or sulphur (0.3%) on salicylic acid (**A**), and indole butyric acid (**B**) of Balady mandarin fruits during Off year. Data were presented as means (*n* = 3 ± SE).

The presented data (Fig. 6A) indicated that all treatments increased salicylic acid levels compared to the control treatment. Also, the highest salicylic acid levels were recorded in KC + M followed by KN + M which recorded highest salicylic acid content by 42.9 and 37.5% compared to the control. Salicylic acid necessary for flower formation^33,34^ flower development^35^, flower longevity^36^, extended flowering period^33^, pollen germination^74,75^, pollen tube elongation^74,75^ and development of ovary after fertilization^76–78^. Specially under stressed condition^79,80^. Moreover, SA enhancing the flower longevity by increasing antioxidant system activity (superoxide dismutase, catalase and ascorbate peroxidase), thereby retarding the senescence of cut N. plumbaginifolia flowers^36^. In this study heavy crop during On year make a stress which consumption plant nutrients and carbohydrates which stimulate accumulation and activity of SA as flowering stimulators^79,80^. Also, it may be increase flowering and pollination period which increase fruit set in the Off year.

The presented data (Fig. 6B) demonstrated that all treatments increased indole butyric acid levels compared to the control treatment. Also, the highest indole butyric acid levels were recorded by KC + S followed by KN + S then KC + M which recorded highest indole butyric acid by 89.36, 85.1 and 40% compared to the control. Auxin succeeded in decreasing the accumulation of reactive oxygen species and extended the ornamental period of lotus flower^33^. Inhibition of inflorescence growth due to fruit load is associated with a selective dampening of auxin transport in the apical region of the stem^37^. Following pollination, auxin levels rise in developing seeds, which is essential for initiating the change from ovary to fruit development^78^. Auxin encourages cell enlargement and division during fruit development^81–84^.

During On year, more fruit depleted the nutrients and carbohydrates from the trees. Also, the seed in the fruits supply with more and more gibberellins during winter, the time of flower bud induction, subsequently it decreased the flowering and then fruiting in the following Off year^6,31,32^. While, PC + M accelerate fruit harvesting with more than 20 days, which means stopped the supply of gibberellins due to fruit removal in harvesting. Also, this materials improved the quality massive yield during On year with more fruit competition. Moreover, this materials specially M produce ethylene which consider one of the flowering stimulate hormones during flower bud induction. Furthermore, K, S and M treatments specially PC + M increased salicylic acid and auxin which improve flowering in the following Off year and decrease plant stress which resulting from more nutrients and carbohydrates depletion in On year. Also, salicylic acid extend flower long-life period and improve pollen germination and increased fruit set^33–36^. Auxin encourages cell enlargement and division during fruit development^37,79,81,83,84^.

The interaction hormonal regulated effects of reducing AB during this study may explained as following. During On year, K and M mainly followed by K + S accelerates harvest, which stopped releasing of GA from seeds by removing fruit and early stopped for carbohydrate depletion. Also, during On year, at flower bud induction time, treatment of M acting as anti-gibberellins which stimulate it and resulting in increased flower buds induction, which led to greater flowering in the subsequent Off season. Additionally, in the Off year, a rise in auxins and salicylic acid levels during flowering enhances flower longevity, pollination effectiveness which increased fruit set and yield.

## Conclusion

Foliar nourishment with KN or KC combined with M or S improve yield, fruit quality and alleviating (AB) alternate bearing in Balady mandarin trees. The most promising treatments is KC combined with M which recorded the highest fruit quality (fruit weight, TSS, TSS/Acid ratio, total sugars) and fruit yield by 20.72% and 33.74% during On year. Furthermore, during Off year, it reduced gibberellins levels in leaves by 19.4%&17.44%, enhancing fruit numbers by 272.64 & 267.94% and yields by 251.3% & 289.65%. The findings suggest that KC combined with methionine M is key for improving fruit quality during heavy load years and aids in managing AB effectively in the following Off year.

## Data Availability

The data generated and/or analysed during the current study are available per request to the corresponding author.
